# Self-esteem and health indicators in Polish postmenopausal women

**DOI:** 10.3389/fnut.2025.1599776

**Published:** 2025-08-26

**Authors:** Karolina Krupa-Kotara, Agnieszka Gdańska, Mateusz Grajek, Tuğrul Günay, Mateusz Rozmiarek

**Affiliations:** ^1^Department of Epidemiology, Faculty of Public Health in Bytom, Medical University of Silesia, Katowice, Poland; ^2^Cracow Higher School of Health Promotion Having Its Registered Office in Cracow, Health Promotion Faculty, Cracow, Poland; ^3^Department of Public Health, Faculty of Public Health in Bytom, Medical University of Silesia, Katowice, Poland; ^4^Faculty of Tourism, Cyprus Science University, Kyrenia, Türkiye; ^5^Department of Sports Tourism, Faculty of Physical Culture Sciences, Poznan University of Physical Education, Poznan, Poland

**Keywords:** self-esteem, health indicators, physical activity, older women, aging, psychological wellbeing, quality of life, social integration

## Abstract

**Background:**

Aging populations face health and psychosocial challenges that can affect their quality of life. This study aimed to examine the relationship between self-esteem and selected health indicators among older women in Poland.

**Methods:**

Six hundred women aged 60+ attending Third Age Universities were surveyed. BMI, waist-to-hip ratio (WHR), daily physical activity (steps), fasting glucose and cholesterol levels, and self-esteem (Rosenberg Self-Esteem Scale) were assessed.

**Results:**

Adverse health indicators such as high BMI, WHR, and elevated glucose were associated with lower self-esteem (*p* < 0.05). Cholesterol levels showed no significant relationship.

**Conclusion:**

These findings suggest that maintaining a healthy lifestyle and metabolic balance may contribute to better psychological wellbeing for older women. Community-based physical activity initiatives may support psychological wellbeing among postmenopausal women.

## 1 Introduction

Demographic changes associated with an aging population are becoming increasingly important. Projections indicate that the proportion of individuals over the age of 65 in Poland will steadily rise, posing challenges to health systems and social support structures (1). In response, the World Health Organization (WHO) recommends that older adults engage in regular physical activity to delay the onset of chronic diseases and maintain independence (2, 3).

Physical activity has been shown to positively influence musculoskeletal, cardiovascular, and cognitive health in older adults (4, 5). It helps prevent functional decline, supports mobility, and reduces the risk of conditions such as osteoporosis, hypertension, diabetes, and depression (6–9). The WHO guidelines emphasize the importance of maintaining movement even in older age to support long-term health (10–13).

Regular exercise also promotes emotional and social wellbeing by enhancing mood and facilitating interpersonal contact (14, 15). Activities such as dance or Nordic walking contribute not only to physical fitness but also to social integration, which helps combat loneliness, a key risk factor for depression in older age (16, 17). However, many older adults remain inactive due to barriers such as health limitations, fear of injury, or lack of access to tailored programs (18).

Despite extensive research on self-esteem across the lifespan, studies specifically examining the influence of physical and metabolic health indicators on self-esteem in postmenopausal women are scarce. Self-esteem is widely recognized as a multidimensional construct comprising both trait and state aspects (19). Trait self-esteem reflects general, stable self-worth, while state self-esteem is sensitive to situational factors, including health and appearance. Self-esteem is influenced by perceived body image and social attitudes (20) and may be particularly vulnerable to age- and weight-related stigma (21).

This study aims to explore the relationship between self-esteem and selected health indicators, body mass index (BMI), waist-to-hip ratio (WHR), physical activity, glucose, and cholesterol levels in a sample of postmenopausal women in Poland. Understanding these associations may help inform future psychological and behavioral interventions that promote wellbeing in older populations.

## 2 Materials and methods

The study was conducted on a group of 600 older women, students of Third Age Universities in the Silesian Voivodeship in Poland, to identify the relationships between self-esteem levels and health indicators such as BMI, WHR, physical activity, glucose levels, and cholesterol levels. Participants were recruited from both independent living individuals and those residing in care homes or attending senior activity programs. Inclusion criteria encompassed individuals over the age of 60 without severe cognitive impairments or acute health failure. In contrast, individuals with advanced neurodegenerative diseases, severe depressive disorders, or who did not provide informed consent were excluded from the study.

Data collection was carried out using self-assessment questionnaires and anthropometric measurements. The key research tool was the Rosenberg Self-Esteem Scale (RSES), which allowed for the assessment of participants’ self-esteem levels. The Rosenberg Self-Esteem Scale (RSES) was originally developed by Rosenberg in 1965 and is widely used across diverse populations. For this study, the Polish adaptation by Łaguna et al. (22) was used. The scale showed good internal consistency in the current sample (Cronbach’s alpha = 0.86), indicating satisfactory reliability for further analyses. This scale consists of 10 questions, with scores ranging from 10 to 40 points, where higher values indicate better self-evaluation. The cutoff values used to categorize self-esteem levels (low ≤25, medium 26–35, high ≥36) were based on normative recommendations provided in the Polish adaptation by Łaguna et al.

Anthropometric measurements included body mass index (BMI) and waist-to-hip ratio (WHR). BMI was calculated as the ratio of body weight to the square of height and then categorized into six groups: underweight, normal weight, overweight, and three degrees of obesity. WHR was determined as the ratio of waist circumference to hip circumference, with individuals having a value of ≥0.85 classified as having central obesity. The anthropometric measurements used in this study (BMI and WHR) follow World Health Organization (WHO) guidelines and classification standards (23).

Physical activity levels were assessed based on the number of steps taken by participants throughout the week, using pedometers worn for 7 days. Participants taking fewer than 5,000 steps per day were classified as low-active, those taking between 5,000 and 10,000 steps belonged to the moderate-activity group, while individuals exceeding 10,000 steps daily were categorized as highly active. The classification of physical activity levels based on daily step counts follows Tudor-Locke et al. (24).

The study also included laboratory analysis of glucose and cholesterol levels. Fasting glucose levels were measured and classified into four categories: hypoglycemia (≤70 mg/dL), normal (71–90 mg/dL), elevated (91–125 mg/dL), and diabetes (>125 mg/dL). Total cholesterol was divided into three categories: normal (<200 mg/dL), elevated (200–239 mg/dL), and high (≥240 mg/dL). Fasting glucose and cholesterol levels were measured using standard laboratory methods with the Cobas Integra 400 Plus analyzer (Roche Diagnostics). Anthropometric measurements were taken using a calibrated electronic scale and a non-elastic measuring tape.

The term ‘health indicators’ in this study refers to commonly used anthropometric and metabolic measures (BMI, WHR, glucose, cholesterol) recommended by the World Health Organization as markers of health status in epidemiological research (23).

The study was conducted in accordance with scientific ethical principles. Each participant was informed about the purpose of the study, data collection methods, and the analysis process. Informed consent was required for participation, and data were appropriately coded to ensure anonymity. Moreover, participants have the right to withdraw from the study at any time without providing a reason. The research procedure complied with the principles of the Declaration of Helsinki and was subject to the supervision of a bioethics committee where required. The specific approval ID for this research is PCN/CBN/0052/KB/127/22, which can be referenced for further verification and transparency.

Statistical analysis was performed to determine the relationships between variables. Percentage distributions were calculated to define the proportion of each category within the studied group. To assess the relationships between self-esteem and other variables, the chi-square test was applied to determine the statistical significance of differences between categories. In addition to the chi-square test, Pearson’s correlation coefficients were calculated to assess the strength and direction of relationships between continuous variables where appropriate. Statistical significance was set at a *p*-value of ≤0.05.

## 3 Results

The study conducted among 600 women aged 60 to 75, participants of Third Age Universities in the Silesian Voivodeship, allowed for a detailed analysis of their health status, lifestyle, and self-esteem (Table 1).

**Table 1:** Demographic characteristics of participants (*N* = 600).

Variable	Category	*N* (%)
Age (mean ± SD)		67.4 ± 5.2
Education	Primary	116 (19.3%)
	Secondary	298 (49.7%)
	Tertiary	186 (31.0%)
Marital status	Married	237 (39.5%)
	Widowed	269 (44.8%)
	Divorced / Single	94 (15.7%)
Financial status	Good	147 (24.5%)
	Average	362 (60.3%)
	Financial difficulties	91 (15.2%)
Place of residence	Medium-sized city	304 (50.7%)
	Large urban area	176 (29.3%)
	Rural/small town	120 (20.0%)

The respondents represented a diverse educational background; approximately 50% had secondary education, 30% had higher education, and 20% had vocational or primary education. Self-esteem scores differed by education level (*p* = 0.03), with higher scores among those with higher education. Regarding family status, the majority were widows (approximately 45%) or married (40%), while 15% were divorced or living alone. The professional status of the respondents was predictable for this age group: 85% were retired, 10% remained professionally active in a limited capacity (e.g., part-time work and self-employment), and 5% identified as unemployed without pension benefits.

In terms of their financial situation, participants provided varied assessments. The majority, approximately 60%, described their financial status as average, sufficient to cover daily expenses but without significant savings. Approximately 25% reported a good financial situation, allowing for additional spending, travel, and recreational activities, while 15% reported financial difficulties, emphasizing the need for saving and expenditure reduction.

Regarding place of residence, women from medium-sized cities (up to 100,000 inhabitants) dominated the study group, accounting for 50% of the sample. Approximately 30% lived in large urban areas, while the remaining 20% resided in rural areas and small towns.

Analyzing the self-esteem level of participants based on the Rosenberg Scale, it was observed that 44.7% had average self-esteem, indicating moderate self-worth and self-acceptance. High self-esteem was reported by 30% of the women, while 25.3% had low self-esteem, suggesting a greater tendency toward self-criticism and lower satisfaction with personal achievements and appearance.

**Figure 1:**
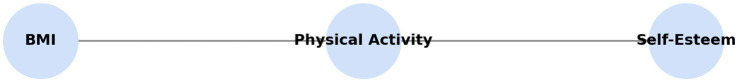
Path diagram illustrates hypothesized relationships between body mass index (BMI), physical activity, and self-esteem. The model assumes that BMI influences both physical activity levels and self-esteem directly, while physical activity mediates part of this relationship. This conceptual framework provides a basis for interpreting the associations observed in regression analysis.

The hypothesized relationships between body mass, physical activity, and self-esteem are summarized in Figure 1.

The body mass index (BMI) results showed that only 27.3% of women were within the normal range, whereas the majority were overweight (48%) or obese to varying degrees (a total of 24%). Among them, 16% had first-degree obesity, 5.3% had second-degree obesity, and 2.7% had third-degree obesity. Only 0.7% were underweight. Additionally, the waist-to-hip ratio (WHR) analysis indicated that 72% of the participants had values exceeding 0.85, which may suggest an increased risk of metabolic and cardiovascular diseases.

The participants’ physical activity levels were not high; approximately half (48%) exhibited low physical activity, 44% had moderate activity, and only 8% regularly engaged in more intense physical exercise.

Regarding blood glucose levels, 40% of women were within the normal range (71–90 mg/dL), but another 40% had elevated levels (91–125 mg/dL), indicating an increased risk of developing diabetes. Moreover, 10% of participants were already diagnosed with diabetes (glucose levels above 125 mg/dL), while another 10% had hypoglycemia (≤70 mg/dL).

A similar trend was observed for cholesterol levels: 40% of women had normal levels (<200 mg/dL), 40% had elevated levels (200–239 mg/dL), and 20% had high cholesterol levels (≥240 mg/dL), potentially increasing the risk of cardiovascular diseases.

Further analysis examined the relationship between wellbeing, measured by the Rosenberg Scale, and selected variables.

The findings indicate a significant correlation between BMI and self-esteem (*p* = 0.032). Women with overweight and obesity were more likely to report lower self-esteem. Among those with second- and third-degree obesity, approximately 70% had low self-esteem, while only 10% had high self-esteem. In contrast, in the group at normal weight, as many as 40% declared high self-esteem. Women with underweight (0.7% of the group) also exhibited lower self-esteem.

A significant correlation was also observed between WHR and self-esteem (*p* = 0.041). Among those with WHR above 0.85, 60% reported low or average self-esteem, while only 15% had high self-esteem. Conversely, in the group with a lower WHR (28% of participants), 45% had high self-esteem.

**Figure 2:**
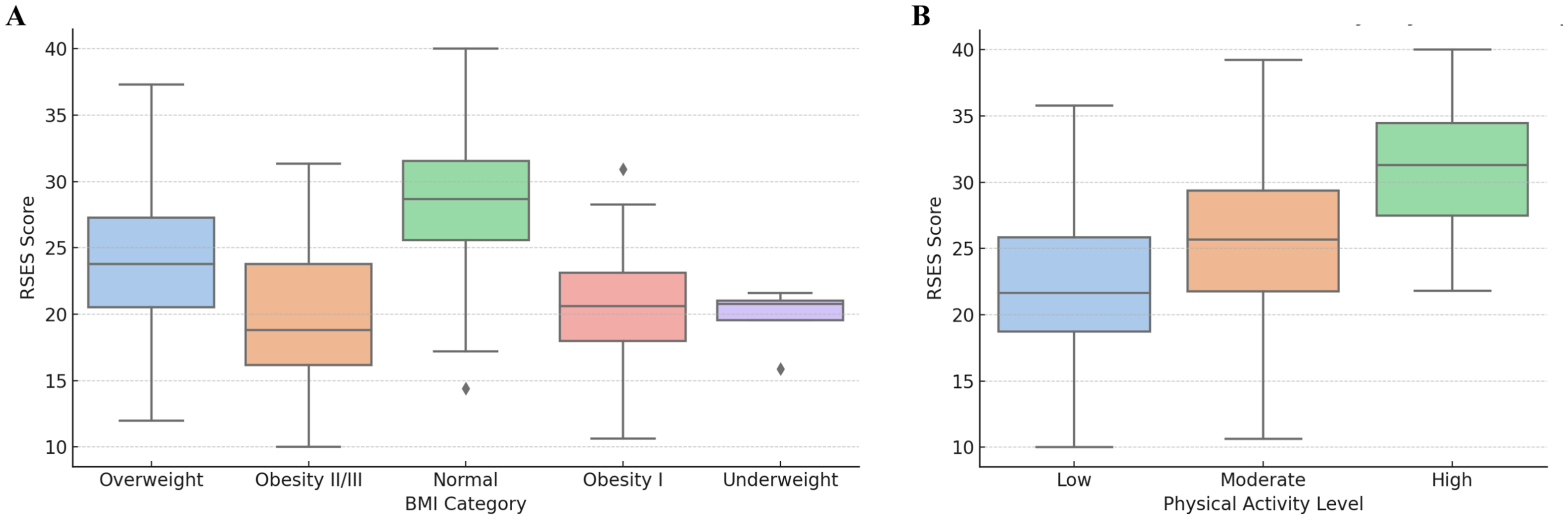
**(A)** Boxplot shows Rosenberg Self-Esteem Scale (RSES) scores across the BMI categories. Participants with normal weight demonstrated the highest median self-esteem scores, while underweight and obese individuals tended to report lower values. **(B)** Boxplot presents the RSES scores across the levels of physical activity. Self-esteem was lowest among women with low physical activity and highest among those with high daily movement, suggesting a positive association between physical activity and psychological wellbeing.

The strongest correlation was found between physical activity levels and self-esteem (*p* = 0.015). The distribution of self-esteem scores across BMI and physical activity categories is presented in Figures 2A,B. Women with high physical activity (8% of participants) had high self-esteem in approximately 80% of cases, whereas among those with low physical activity (48% of participants), 50% reported low self-esteem.

A significant correlation between glucose levels and self-esteem (*p* = 0.028) suggests that individuals with elevated glucose and diabetes more frequently have low self-esteem. Among those with diabetes, 70% had low self-esteem, whereas in the group with normal glucose levels, only 20% had low self-esteem, and as many as 50% had high self-esteem.

In contrast to glucose, the analysis did not reveal a significant correlation between cholesterol levels and self-esteem (*p* = 0.423). Regardless of whether cholesterol levels were normal, elevated, or high, no substantial differences in self-esteem levels were observed (Table 2).

**Table 2:** Study variables and their impact on participants’ self-esteem.

Variable	Categories	% Of people in each category	*χ* ^2^	*p*-value
BMI	Underweight	0.70%	7.25	0.032
	Normal	27.30%		
	Overweight	48%		
	Obesity I	16%		
	Obesity II	5.30%		
	Obesity III	2.70%		
WHR	WHR ≥ 0.85	72%	6.5	0.041
	WHR < 0.85	28%		
Physical activity	Low physical activity	48%	8.1	0.015
	Moderate physical activity	44%		
	High physical activity	8%		
Glucose	Hypoglycemia (≤70 mg/dL)	10%	14.8	0.028
	Normal (71–90 mg/dL)	40%		
	Elevated (91–125 mg/dL)	40%		
	Diabetes (>125 mg/dL)	10%		
Cholesterol	Normal (<200 mg/dL)	40%	3.88	0.423
	Elevated (200–239 mg/dL)	40%		
	High (≥240 mg/dL)	20%		

Logistic regression analysis (Table 3) indicated that low self-esteem was significantly associated with higher BMI, central obesity (WHR ≥ 0.85), low physical activity, elevated glucose levels, and low socioeconomic status (SES). The strongest effect was observed for physical activity, where low activity increased the odds of low self-esteem more than 2-fold (OR = 2.10, *p* = 0.005).

**Table 3:** Logistic regression model predicts low self-esteem based on selected health indicators.

Variable	OR (95% CI)	*p*-value
BMI (per category)	1.42 (1.10, 1.85)	0.012
WHR ≥ 0.85	1.36 (1.01, 1.82)	0.044
Low PA vs. High PA	2.10 (1.25, 3.52)	0.005
Elevated glucose (91–125 mg/dL)	1.28 (1.02, 1.60)	0.031
Diabetes (>125 mg/dL)	1.68 (1.09, 2.56)	0.018
Low SES	1.54 (1.02, 2.30)	0.037

**Figure 3:**
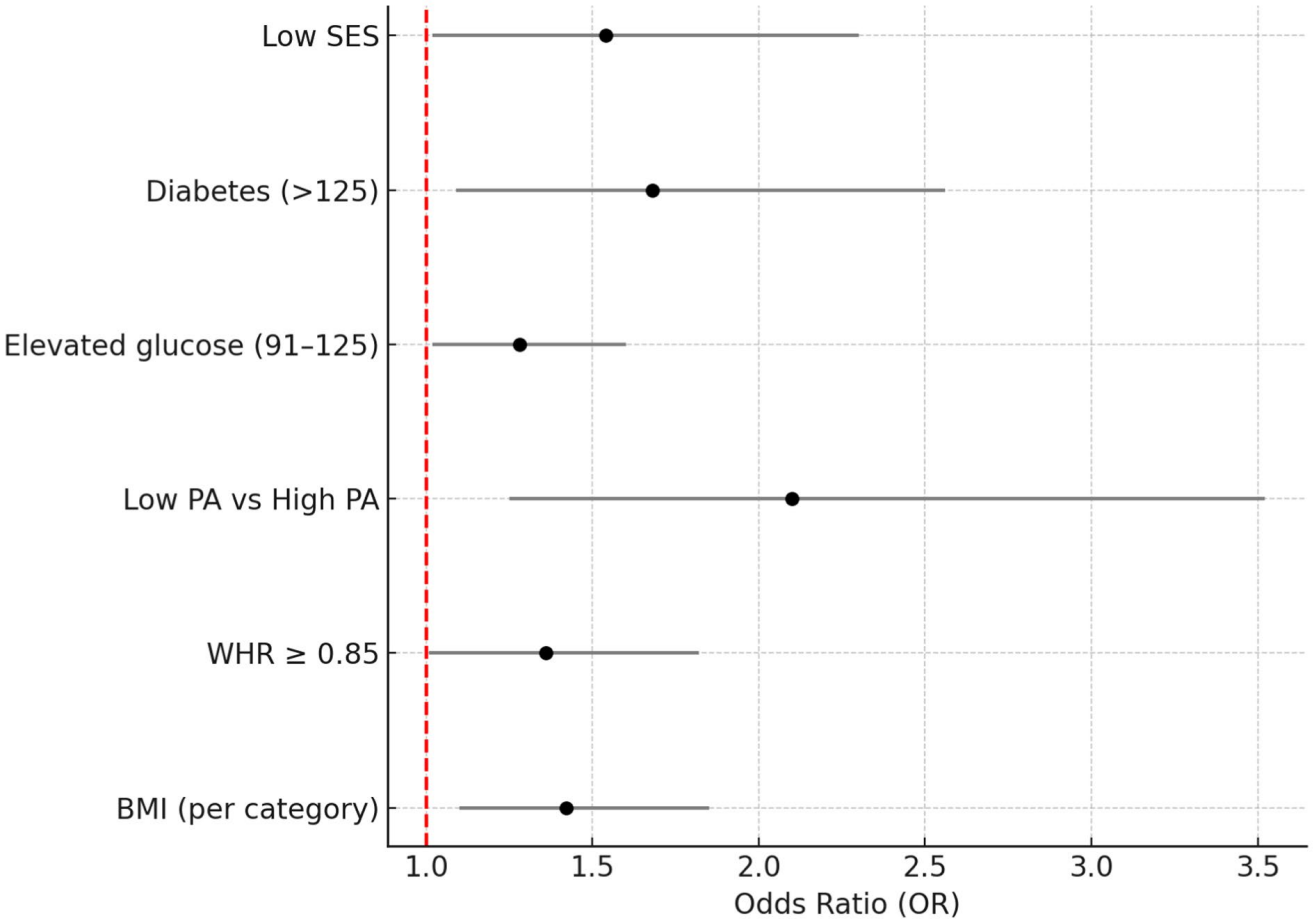
Forest plot presents adjusted odds ratios (ORs) and 95% confidence intervals for the relationship between low self-esteem and selected health indicators (BMI, WHR, physical activity, glucose, and SES). The vertical red line indicates no effect (OR = 1).

The regression results are visualized in Figure 3, which shows adjusted odds ratios with 95% confidence intervals for each predictor variable.

## 4 Discussion

This study investigated the relationships between self-esteem and selected health indicators in a sample of older women. The main findings indicate that higher BMI, higher WHR, lower physical activity, and elevated glucose are associated with lower self-esteem, while cholesterol levels did not show a significant relationship. These results are discussed below in relation to existing literature and practical implications. The primary objective of this study was to determine the relationship between self-esteem levels and health indicators such as BMI, WHR, physical activity, and glucose and cholesterol levels among older women attending Universities of the Third Age in the Silesian Voivodeship. The study aimed to analyze the impact of health-related and lifestyle factors on the subjective perception of self-worth among women over the age of 60.

The findings indicated a significant association between BMI and self-esteem (*p* = 0.032). Older adults with overweight and obesity were more likely to report lower self-esteem, which aligns with studies conducted by Adam et al. (25) and reflects a trend observed in children and adults, as noted in the reviews by French et al. (26) and Griffiths et al. (27). This pattern is consistent with findings that higher central adiposity is linked to poorer body image and lower self-esteem among women (28). Among individuals with grade II and III obesity, approximately 70% exhibited low self-esteem, whereas only 10% reported high self-esteem. In contrast, among those with a normal body weight, as many as 40% declared high self-esteem. Furthermore, underweight individuals (0.7% of the group) also demonstrated reduced self-esteem, suggesting that both excessive and insufficient body weight may negatively impact self-perception.

As the proportion of individuals over the age of 65 increases in the population, the prevalence of overweight and obesity is also rising (29, 30). This phenomenon poses significant public health concerns, as obesity is associated with an increased risk of cardiovascular diseases, diabetes, neurodegenerative disorders, and higher mortality rates. Moreover, as demonstrated by Tolppanen et al. (31), regular physical activity may play a crucial role in reducing the risk of dementia and other neurodegenerative conditions, which are often comorbid with overweight in older age. Excess weight can also contribute to a decline in quality of life and reduced independence, which may further translate into lower self-esteem. Another critical issue in obesity management among older adults is the risk of sarcopenic obesity, characterized by a low proportion of lean body mass, leading to reduced physical fitness and diminished self-worth (32–34).

This study also found a significant association between WHR and self-esteem (*p* = 0.041). Among individuals with WHR above 0.85, 60% reported low or moderate self-esteem, while only 15% had high self-esteem. Conversely, in the group with lower WHR (28% of the participants), 45% exhibited high self-esteem. This suggests that not only body weight, but also fat distribution affects self-perception; individuals with a more central type of obesity may be more prone to experiencing negative emotions related to their appearance. The association between WHR and self-esteem may be explained by body image dissatisfaction and social appearance anxiety, which are known to increase with central adiposity, particularly among older women. Research shows that concerns about physical appearance remain significant even in late adulthood, potentially affecting self-esteem and social participation. Unlike general overweight, central obesity may be more visible and socially stigmatized, particularly in older women, amplifying its psychological impact.

The strongest association observed in this study was between physical activity levels and self-esteem (*p* = 0.015). Among individuals with high physical activity levels (8% of participants), approximately 80% reported high self-esteem, confirming previous findings by McAuley et al. (35) and Elavsky et al. (36). In contrast, in the low-activity group (48% of participants), as many as 50% reported low self-esteem. Regular physical activity may enhance self-esteem by supporting physical fitness, promoting overall wellbeing, and encouraging independence in daily functioning. Numerous studies support the beneficial effects of physical activity on health. For example, Kraus et al. (37) demonstrated that aerobic exercises such as walking and running can effectively improve lipid profiles, further contributing to better wellbeing and greater body acceptance.

Regular physical exercise is one of the most effective methods of counteracting adverse changes associated with aging (38–40). It can reduce the risk of developing various diseases, including diabetes, lipid disorders, cardiovascular diseases, and cancer. Moreover, as Ballin et al. (10) demonstrated, even a short 10-week high-intensity interval training (HIIT) program can significantly reduce total cholesterol (TC) and LDL-C levels in older individuals with obesity. These findings highlight the importance of physical activity for both metabolic and psychological health.

This study also identified a significant association between glucose levels and self-esteem (*p* = 0.028). Individuals with elevated glucose levels and diabetes were more likely to have low self-esteem. Among participants with diabetes, as many as 70% reported low self-esteem, whereas in the group with normal glucose levels, only 20% reported low self-esteem, while 50% reported high self-esteem. This may be attributed to the impact of diabetes on overall quality of life and wellbeing. Chronic fatigue, weakness, and complications may contribute to reduced self-worth. While both glucose and cholesterol levels are relevant health indicators, glucose levels may have a more direct impact on daily functioning due to symptoms such as fatigue, dizziness, or neuropathy, which are tangible to the individual. In contrast, elevated cholesterol often remains asymptomatic, making it less likely to influence subjective self-esteem. The lack of a significant relationship between cholesterol levels and self-esteem may be partly explained by the nature of cholesterol as a ‘silent’ risk factor; it often does not produce noticeable physical symptoms and may be less salient in daily self-perception compared to weight or glucose-related fatigue. Previous studies indicate that awareness of metabolic risks, such as dyslipidemia, tends to be lower than for conditions such as diabetes or obesity, which could explain the weak link with self-esteem in this study.

In contrast to glucose, the analysis did not reveal a significant association between cholesterol levels and self-esteem (*p* = 0.423). The lack of significant awareness of cholesterol status may limit its influence on self-perception. Since cholesterol is not a directly perceptible health indicator, many individuals may be unaware of their cholesterol levels, which means it does not influence self-esteem to the same extent as body weight or physical activity. The results confirm that promoting regular physical activity may significantly enhance both the physical and psychological dimensions of health-related quality of life among physically active individuals (41). The results of the present study are consistent with our previous findings (42), which indicate that a higher level of physical activity contributes to higher global self-esteem and better health indicators, also among younger population groups.

Weight-related stigma remains prevalent among older adults, potentially affecting self-perception and psychological health (20).

Finally, the observed relationships should be interpreted in the context of traditional gender roles. Older women may internalize societal standards of appearance and caregiving responsibilities more strongly, which can shape both health behaviors and self-esteem. Acknowledging these gendered expectations is essential for designing effective interventions to support psychological wellbeing in older women.

## 5 Study limitations

Despite its significant findings, this study has several limitations that should be considered when interpreting the results. First, the study sample consisted exclusively of older women attending Universities of the Third Age in the Silesian Voivodeship, which limits the generalizability of the findings to the broader elderly population, particularly those not engaged in similar educational and social activities. This group’s higher education level and engagement may bias the results upward compared to the general postmenopausal population. Additionally, the cross-sectional nature of the study prevents the determination of causal relationships between the analyzed variables. It remains unclear whether low self-esteem results from being overweight and a lack of physical activity or whether low self-esteem leads to reduced health-conscious behaviors. As this study is cross-sectional and based on correlations, causality cannot be inferred, and the observed associations should be interpreted with caution.

Another limitation is the use of self-report questionnaires, such as the Rosenberg Self-Esteem Scale (RSES), which may be subject to biases, including a tendency to present oneself in a more favorable light. Similarly, while the assessment of physical activity using pedometers provides objective data, it may not fully capture overall activity levels, as it does not account for exercise intensity or other forms of movement, such as strength training or group exercise sessions. Furthermore, the analysis of glucose and cholesterol levels was based on a single measurement, which does not allow for an evaluation of changes in these parameters over time. As measurements were taken at a single time point, temporal variability in health indicators could not be assessed.

Another potential limitation is the lack of data on other psychological and social factors that may influence self-esteem, such as social support, a history of depression, or life circumstances. Despite these limitations, this study provides valuable insights into the relationship between health status and self-esteem among older women, emphasizing the importance of health prevention and an active lifestyle in promoting psychological wellbeing. Future research should include depression and socioeconomic status (SES) as covariates, given their potential role in mediating the relationship between health status and self-esteem.

Moreover, it should be noted that the study sample consisted exclusively of older women enrolled in Third Age University (TAU) programs in the Silesian Voivodeship. This group may differ from the broader older adult population in terms of socio-economic status, higher educational engagement, and greater health awareness, which could limit the generalizability of the findings to less active or more socially isolated older adults.

Future research could benefit from supplementing chi-square analyses with parametric tests (e.g., t-tests and ANOVA) where assumptions of normality are met, providing deeper insights into mean differences between groups.

## 6 Conclusion

Both body weight and body proportion play a crucial role in shaping self-esteem. Individuals with overweight and obesity were more likely to report lower self-esteem, whereas those with more balanced body proportions tended to assess themselves more positively. Physical activity may be associated with greater self-esteem among older women, but further research is needed to confirm causality, suggesting that programs promoting movement, especially among older adults, may positively influence their self-perception and overall quality of life. Conversely, diabetes was significantly associated with lower self-esteem, highlighting the importance of health prevention and effective disease management in the context of psychological wellbeing. Strategies should include community-based physical activity programs tailored to older women, promoting body confidence and health. In contrast, cholesterol levels did not have a significant impact on self-esteem, indicating that not all metabolic indicators translate into subjective self-perception. Future longitudinal studies are needed to confirm the causal nature of these associations.

## Data Availability

The original contributions presented in the study are included in the article/supplementary material, further inquiries can be directed to the corresponding author.

## References

[1] Chavez A Scales R Kling JM. Promoting physical activity in older women to maximize health. Cleve Clin J Med. (2021) 88:405–15. doi: 10.3949/ccjm.88a.20170, PMID: 34210715

[2] Baill IC Castiglioni A. Health maintenance in postmenopausal women. Am Fam Physician. (2017) 95:561–70. 28671391

[3] Riedl I Yoshioka M Nishida Y Tobina T Paradis R Shono N. Regulation of skeletal muscle transcriptome in elderly men after 6 weeks of endurance training at lactate threshold intensity. Exp Gerontol. (2010) 45:896–903. doi: 10.1016/j.exger.2010.08.014, PMID: 20813182

[4] Sabia S Nabi H Kivimaki M Shipley MJ Marmot MG Singh-Manoux A. Health behaviors from early to late midlife as predictors of cognitive function. Am J Epidemiol. (2009) 170:428–37. doi: 10.1093/aje/kwp161 19574344 PMC2727179

[5] Pinheiro MB Oliveira J Bauman A Fairhall N Kwok W Sherrington C. Evidence on physical activity and osteoporosis prevention for people aged 65+ years: a systematic review to inform the WHO guidelines on physical activity and sedentary behaviour. Int J Behav Nutr Phys Act. (2020) 17:150. doi: 10.1186/s12966-020-01040-4, PMID: 33239014 PMC7690138

[6] Krenn M Haller M Bijak M Unger E Hofer C Kern H. Safe neuromuscular electrical stimulator designed for the elderly. Artif Organs. (2011) 35:253–6. doi: 10.1111/j.1525-1594.2011.01217.x, PMID: 21401669

[7] Iso-Markku P Kujala UM Knittle K Polet J Vuoksimaa E Waller K. Physical activity as a protective factor for dementia and Alzheimer’s disease: systematic review, meta-analysis and quality assessment of cohort and case-control studies. Br J Sports Med. (2022) 56:701–9. doi: 10.1136/bjsports-2021-104981, PMID: 35301183 PMC9163715

[8] Stringhini S Carmeli C Jokela M Avendaño M Muennig P Guida F. Socioeconomic status and the 25 × 25 risk factors as determinants of premature mortality: a multicohort study and meta-analysis of 1.7 million men and women. Lancet. (2017) 389:1229–37. doi: 10.1016/S0140-6736(16)32380-7, PMID: 28159391 PMC5368415

[9] Ungvari Z Fazekas-Pongor V Csiszar A Kunutsor SK. The multifaceted benefits of walking for healthy aging: from blue zones to molecular mechanisms. Geroscience. (2023) 45:3211–39. doi: 10.1007/s11357-023-00873-8, PMID: 37495893 PMC10643563

[10] Ballin M Lundberg E Sörlén N Nordström P Hult A Nordström A. Effects of interval training on quality of life and cardiometabolic risk markers in older adults: a randomized controlled trial. Clin Interv Aging. (2019) 14:1589–99. doi: 10.2147/CIA.S213133, PMID: 31564841 PMC6732517

[11] Bull FC Al-Ansari SS Biddle S Borodulin K Buman MP Cardon G. World Health Organization 2020 guidelines on physical activity and sedentary behaviour. Br J Sports Med. (2020) 54:1451–62. doi: 10.1136/bjsports-2020-102955, PMID: 33239350 PMC7719906

[12] Howe TE Shea B Dawson LJ Downie F Murray A Ross C. Exercise for preventing and treating osteoporosis in postmenopausal women. Cochrane Database Syst Rev. (2011) 6:CD000333. doi: 10.1002/14651858.CD000333.pub2 PMC12744941 21735380

[13] Marin-Cascales E Alcaraz PE Ramos-Campo DJ Rubio-Arias JA. Effects of multicomponent training on lean and bone mass in postmenopausal and older women: a systematic review. Menopause. (2018) 25:346–56. doi: 10.1097/GME.0000000000000975, PMID: 28816931

[14] Lee SW Patel R Werner B Yoo JW Tiu T. The role of physical activity in older adults and practical intervention. HCA Healthc J Med. (2021) 2:387–95. doi: 10.36518/2689-0216.1352, PMID: 37427399 PMC10324802

[15] Santos DPMA Queiroz ACDCM Menezes RL Bachion MM. Effectiveness of senior dance in the health of adults and elderly people: an integrative literature review. Geriatr Nurs. (2020) 41:589–99. doi: 10.1016/j.gerinurse.2020.03.013, PMID: 32354477

[16] Lee HS Park JH. Effects of Nordic walking on physical functions and depression in frail people aged 70 years and above. J Phys Ther Sci. (2015) 27:2453–6. doi: 10.1589/jpts.27.2453, PMID: 26357424 PMC4563288

[17] O'Brien E. Planning for population ageing: ensuring enabling and supportive physical-social environments—local infrastructure challenges. Planning Theory Pract. (2014) 15:220–34. doi: 10.1080/14649357.2014.902986

[18] Levinger P Sales M Polman R Haines T Dow B Biddle SJH. Outdoor physical activity for older people—the senior exercise park: current research, challenges and future directions. Health Promot J Austr. (2018) 29:353–9. doi: 10.1002/hpja.60, PMID: 29537618

[19] Heatherton TF Polivy J. Development and validation of a scale for measuring state self-esteem. J Pers Soc Psychol. (1991) 60:895–910. doi: 10.1037/0022-3514.60.6.895

[20] Robins RW Trzesniewski KH Tracy JL Gosling SD Potter J. Global self-esteem across the life span. Psychol Aging. (2002) 17:423–34. doi: 10.1037/0882-7974.17.3.423, PMID: 12243384

[21] Puhl RM Heuer CA. The stigma of obesity: a review and update. Obesity (Silver Spring). (2009) 17:941–64. doi: 10.1038/oby.2008.636, PMID: 19165161

[22] Łaguna M Lachowicz-Tabaczek K Dzwonkowska I. Skala samooceny SES Morrisa Rosenberga – polska adaptacja metody. Soc Psychol Społeczna. (2007) 2:164–76.

[23] WHO: World Health Organization. Obesity: Preventing and managing the global epidemic. WHO Technical Report Series No. 894. Geneva: WHO (2000). 11234459

[24] Tudor-Locke C Bassett DR. How many steps/day are enough? Sports Med. (2004) 34:1–8. doi: 10.2165/00007256-200434010-00001, PMID: 14715035

[25] Adam MY Wani MA. Self-esteem and mental health among obese and non-obese people. Int J Health Sci. (2022) 6:1689–705. doi: 10.53730/ijhs.v6nS8.11511

[26] French SA Story M Perry CL. Self-esteem and obesity in children and adolescents: a literature review. Obes Res. (1995) 3:479–90. doi: 10.1002/j.1550-8528.1995.tb00179.x, PMID: 8521169

[27] Griffiths LJ Parsons TJ Hill AJ. Self-esteem and quality of life in obese children and adolescents: a systematic review. Int J Pediatr Obes. (2010) 5:282–304. doi: 10.3109/17477160903473697, PMID: 20210677

[28] Satwik R Sinha D Tiwari B. Prevalence of poor body image and its correlation with self-esteem and depression in middle-aged women. Climacteric (2024) 27:202–9. doi: 10.1080/13697137.2023.2297876 38231656

[29] Peralta M Ramos M Lipert A Martins J Marques A. Prevalence and trends of overweight and obesity in older adults from 10 European countries from 2005 to 2013. Scand J Public Health. (2018) 46:522–9. doi: 10.1177/1403494818764810, PMID: 29569525

[30] Houston DK Nicklas BJ Zizza CA. Weighty concerns: the growing prevalence of obesity among older adults. J Am Diet Assoc. (2009) 109:1886–95. doi: 10.1016/j.jada.2009.08.014, PMID: 19857630

[31] Tolppanen A-M Solomon A Kulmala J Kåreholt I Ngandu T Rusanen M. Leisure-time physical activity from mid- to late life, body mass index, and risk of dementia. Alzheimers Dement. (2015) 11:434–43. doi: 10.1016/j.jalz.2014.01.008, PMID: 24721528

[32] Batsis JA Villareal DT. Sarcopenic obesity in older adults: Aetiology, epidemiology and treatment strategies. Nat Rev Endocrinol. (2018) 14:513–37. doi: 10.1038/s41574-018-0062-9, PMID: 30065268 PMC6241236

[33] Prado CM Batsis JA Donini LM Gonzalez MC Siervo M. Sarcopenic obesity in older adults: a clinical overview. Nat Rev Endocrinol. (2024) 20:261–77. doi: 10.1038/s41574-023-00943-z, PMID: 38321142 PMC12854800

[34] Gao Q Mei F Shang Y Hu K Chen F Zhao L. Global prevalence of sarcopenic obesity in older adults: a systematic review and meta-analysis. Clin Nutr. (2021) 40:4633–41. doi: 10.1016/j.clnu.2021.06.009, PMID: 34229269

[35] McAuley E Elavsky S Motl RW Konopack JF Hu L Marquez DX. Physical activity, self-efficacy, and self-esteem: longitudinal relationships in older adults. J Gerontol B Psychol Sci Soc Sci. (2005) 60:P268–75. doi: 10.1093/geronb/60.5.P268, PMID: 16131621

[36] Elavsky S McAuley E Motl RW Konopack JF Marquez DX Hu L. Physical activity enhances long-term quality of life in older adults: efficacy, esteem, and affective influences. Ann Behav Med. (2005) 30:138–45. doi: 10.1207/s15324796abm3002_6, PMID: 16173910

[37] Kraus WE Houmard JA Duscha BD Knetzger KJ Wharton MB McCartney JS. Effects of the amount and intensity of exercise on plasma lipoproteins. N Engl J Med. (2002) 347:1483–92. doi: 10.1056/NEJMoa020194, PMID: 12421890

[38] McPhee JS French DP Jackson D Nazroo J Pendleton N Degens H. Physical activity in older age: perspectives for healthy ageing and frailty. Biogerontology. (2016) 17:567–80. doi: 10.1007/s10522-016-9641-0, PMID: 26936444 PMC4889622

[39] León-Guereño P Galindo-Domínguez H Balerdi-Eizmendi E Rozmiarek M Malchrowicz-Mośko E. Motivation behind running among older adult runners. BMC Sports Sci Med Rehabil. (2021) 13:1–10. doi: 10.1186/s13102-021-00366-1 34715913 PMC8555191

[40] Nelson ME Rejeski WJ Blair SN Duncan PW Judge JO King AC. Physical activity and public health in older adults: recommendation from the American College of Sports Medicine and the American Heart Association. Circulation. (2007) 116:1094–105. doi: 10.1161/CIRCULATIONAHA.107.185650, PMID: 17671236

[41] Dębska M Dębski PG Miara A. Samoocena jakości życia związanej ze zdrowiem osób dorosłych regularnie aktywnych fizycznie. Ann Acad Med Siles. (2018) 72:1–5. doi: 10.18794/aams/74294

[42] Krupa-Kotara K Markowski J Gdańska A Grajek M Działach E Szlachta G. Global self-esteem, body composition, and physical activity in polish university students. Nutrients. (2023) 15:3907. doi: 10.3390/nu15183907, PMID: 37764691 PMC10536466

